# Somatic symptom disorder: a scoping review on the empirical evidence of a new diagnosis

**DOI:** 10.1017/S0033291721004177

**Published:** 2022-03

**Authors:** Bernd Löwe, James Levenson, Miriam Depping, Paul Hüsing, Sebastian Kohlmann, Marco Lehmann, Meike Shedden-Mora, Anne Toussaint, Natalie Uhlenbusch, Angelika Weigel

**Affiliations:** 1 Department of Psychosomatic Medicine and Psychotherapy, University Medical Centre Hamburg-Eppendorf, Hamburg, Germany; 2 Department of Psychiatry, Virginia Commonwealth University, Richmond, VA, USA; 3 Department of Psychology, Medical School Hamburg, Hamburg, Germany

**Keywords:** diagnosis, diagnostic and statistical manual of mental disorders, international classification of diseases, review, somatic symptom disorder, somatoform disorders

## Abstract

**Background:**

In 2013, the diagnosis of somatic symptom disorder (SSD) was introduced into the Diagnostic and Statistical Manual of Mental Disorders (DSM-5). This review aims to comprehensively synthesize contemporary evidence related to SSD.

**Methods:**

A scoping review was conducted using PubMed, PsycINFO, and Cochrane Library. The main inclusion criteria were SSD and publication in the English language between 01/2009 and 05/2020. Systematic search terms also included subheadings for the DSM-5 text sections; i.e., diagnostic features, prevalence, development and course, risk and prognostic factors, culture, gender, suicide risk, functional consequences, differential diagnosis, and comorbidity.

**Results:**

Eight hundred and eighty-two articles were identified, of which 59 full texts were included for analysis. Empirical evidence supports the reliability, validity, and clinical utility of SSD diagnostic criteria, but the further specification of the psychological SSD B-criteria criteria seems necessary. General population studies using self-report questionnaires reported mean frequencies for SSD of 12.9% [95% confidence interval (CI) 12.5–13.3%], while prevalence studies based on criterion standard interviews are lacking. SSD was associated with increased functional impairment, decreased quality of life, and high comorbidity with anxiety and depressive disorders. Relevant research gaps remain regarding developmental aspects, risk and prognostic factors, suicide risk as well as culture- and gender-associated issues.

**Conclusions:**

Strengths of the SSD diagnosis are its good reliability, validity, and clinical utility, which substantially improved on its predecessors. SSD characterizes a specific patient population that is significantly impaired both physically and psychologically. However, substantial research gaps exist, e.g., regarding SSD prevalence assessed with criterion standard diagnostic interviews.

## Introduction

In 2013, the American Psychiatric Association (APA) introduced “somatic symptom disorder” (SSD) as a new diagnosis in the Diagnostic and Statistical Manual of Mental Disorders (DSM-5) (American Psychiatric Association, 2013). The DSM-5 diagnosis not only received a new name; its diagnostic criteria also differ radically from somatization disorder which it replaced: Following scientific evidence of over two decades (Kroenke, 2003; Voigt et al., 2012), positive psychological criteria were formulated, i.e. excessive health concerns, and exclusion of potentially underlying medical disorders was no longer required. There are three diagnostic criteria (American Psychiatric Association, 2013): The A-criterion requires one or more distressing or disabling somatic symptoms. The B-criterion requires disproportionate and persistent thoughts about the seriousness of one's symptoms (cognitive dimension), high levels of anxiety about health or symptoms (affective dimension), or excessive energy or time devoted to these symptoms or health concerns (behavioral dimension). The C-criterion specifies that somatic symptoms should persist for over 6 months. SSD also replaced DSM-IV's undifferentiated somatoform disorder, hypochondriasis, and the pain disorders. SSD specifiers with regard to severity, pain, and persistence were introduced (Dimsdale et al., 2013). Patients with severe health anxiety and somatic symptoms are now assigned to SSD and those with solely health anxiety without somatic symptoms to “illness anxiety disorder” (IAD).

DSM-5 explicitly allows SSD to be diagnosed in addition to any comorbid somatic disease, thus avoiding both mind−body dualism and equating medically unexplained with psychogenic. The new criteria also meant to reduce stigmatization: Earlier diagnostic concepts of somatoform disorders in DSM-IV-TR (American Psychiatric Association, 2000) and International Classification of Diseases 10th edition (ICD-10) (World Health Organization, 1992) described affected patients as “difficult,” unable to accept that symptoms are not caused by pathophysiology, and repeatedly requesting medical examinations. After publication, SSD criteria were criticized as imprecise (Mayou, 2014) and overinclusive, risking overdiagnosis (Frances, 2013). In the 11th edition of the International Classification of Diseases, ICD-11 (World Health Organization, 2021), which will take effect in January 2022, the former category of somatoform disorders has also been intensively revised and designated with the term “bodily distress disorder” (BDD). BDD is in large parts similar to SSD; in this respect, it is to be expected that some strengths and weaknesses of SSD will also apply to BDD, for which empirical studies are still missing.

A decade of research on the new SSD diagnosis is now available since the start of the scientific discussion that led to the release of DSM-5. From 2019 to 2021, the APA prepared a text revision of the DSM-5, to which authors of this article contributed as a section editor (JL) and reviser (BL). The literature review for DSM-5-TR was expanded into this scoping review. Its primary aim was to summarize the evidence on the diagnostic criteria of SSD, following the topics addressed in the text sections of the SSD chapter of DSM-5. The second aim was to identify the most relevant research gaps regarding SSD.

## Methods

### Search strategy

A review protocol defining the databases and search terms was drafted by the research team and refined by a research librarian (online Supplemental material). We defined outcome domains based on the subheadings for the specific DSM-5 text sections, i.e. diagnostic features, prevalence, development and course, risk and prognostic factors, culture-related diagnostic issues, gender-related diagnostic issues, suicide risk, functional consequences of SSD, differential diagnosis and comorbidity. Table 1 summarizes general inclusion and exclusion criteria and those specific for each text section. To identify all potentially relevant studies, the bibliographic databases PubMED, PsycINFO and the Cochrane Library for systematic reviews were accessed. Searches were conducted between January and May 2020.

**Table 1.** Inclusion and exclusion criteria for literature search within each DSM-5 SSD text section

	Inclusion criteria	Exclusion criteria
General		
	Manuscripts written in English Manuscripts published in peer-reviewed journals during the last 10 years (01/2009–05/2020) Manuscripts that dealt with the DSM-5 SSD and at least one of the below-mentioned text sections DSM-5 SSD B criteria operationalized either through diagnostic interviews, self-report measures (e.g. symptom measure + SSD-12, WI) or clinical judgment	Study protocols Case studies Studies on questionnaire development Reviews without new data Studies on syndromes other than SSD (e.g. somatoform disorders, functional syndromes, irritable bowel syndrome)
DSM-5 text sections		
Diagnostic features	Any type of study addressing the diagnostic criteria of SSD by presenting or referring to empirical data Any type of study that primarily investigate somatoform disorders or illness anxiety disorder but did so with regard to the new SSD criteria Studies, which aimed to evaluate diagnostic and/or therapeutic interventions, if implications were drawn with regard to the diagnostic features of SSD	
Prevalence	Observational studies, i.e. prospective and retrospective cohort studies, case–control studies, and cross-sectional studies, reporting any point or period prevalence estimates from the general population or any kind of clinical population Any type of study reporting prevalence, frequency or occurrence of somatic symptom disorders. Studies were classified as level one if the report data of representative studies from the general population, and level two for reports on prevalence or frequency in defined populations (e.g. general medicine, other secondary or tertiary care settings or specific patient programs)	Studies with preselected SSD patient groups (where SSD was defined as an inclusion criterion)
Development and course	Any type of study reporting on the etiology and development of SSD in a defined sample Any type of study reporting the particular aspects of SSD in particular age groups such as children, adolescents, adults or older aged people Any type of study reporting on remission and response of SSD in a defined sample	Intervention studies without reference to remission or response
Risk and prognostic factors	Any longitudinal/ prospective study relating to risk factors for SSD Any type of study reporting on prognosis, i.e. the course of the SSD diagnosis and to further associated outcomes like health related quality of life, physical and psychological symptom burden	Pediatric studies and review studies
Culture	Any type of study reporting on cultural aspects in light of SSD (i.e. culture-bound syndrome) in any kind of setting in patients with SSD	
Gender	Any type of study reporting on gender-specific aspects in light of SSD in any kind of setting in patients with SSD	
Suicide risk	Any type of study reporting the prevalence and impact of risk factors for any kind of suicidal thoughts or behaviour (i.e. suicidal thoughts, ideation, attempt, completed suicide) in any kind of setting in patients with SSD. Suicidal thoughts or behaviour could be assessed via self-report or observed outcomes (e.g. attempted suicide)	Studies reporting self-harm without suicidal intention
Functional consequences	Any type of study reporting functional consequences in the defined sample Functional consequences are defined as impact of SSD on health-related physical or mental quality of life, physical functioning, mental functioning, impairment, disability, social functioning, work ability, psychological distress, and ability to participate in relevant activities Any type of study reporting the impact of psychological features of SSD on functional consequences	
Differential diagnosis	Any type of study reporting on SSD and differential diagnosis in any kind of setting	
Comorbidity	Observational studies investigating comorbid mental and physical diseases of SSD or comorbidity of any condition with SSD Any type of study examining associations between self-reported symptoms of SSD and self-reported symptoms of other mental diseases in different population-based and clinical samples	

*Note*. SSD, Somatic symptom disorder; SSD-12, Somatic Symptom Disorder B-criteria Scale; WI, Whitley Index.

### Study selection and data extraction

All identified records were collected in Endnote^x9^ where duplicates were removed. All titles and abstracts were screened for their relevance to the DSM-5 text sections, research questions and eligibility criteria. Selected articles were evaluated based on full text and reviewed by at least two researchers. Disagreements regarding inclusion were discussed and a consensus was resolved through team discussions. Reviewers also checked reference lists of studies meeting inclusion criteria, relevant review articles and editorials to identify further relevant studies. For each DSM-5 text section on SSD, searches were conducted separately, and the number of identified studies was documented. Subsequently, key information within each DSM-5 text section was extracted into a standard data form including publication year, study population, study design, SSD assessment and DSM-5 text section. Review findings are reported according to the Preferred Reporting Items for Systematic Reviews and Meta-Analysis extension for Scoping Reviews (PRISMA-ScR) statement (Tricco et al., 2018).

## Results

**Fig. 1.**
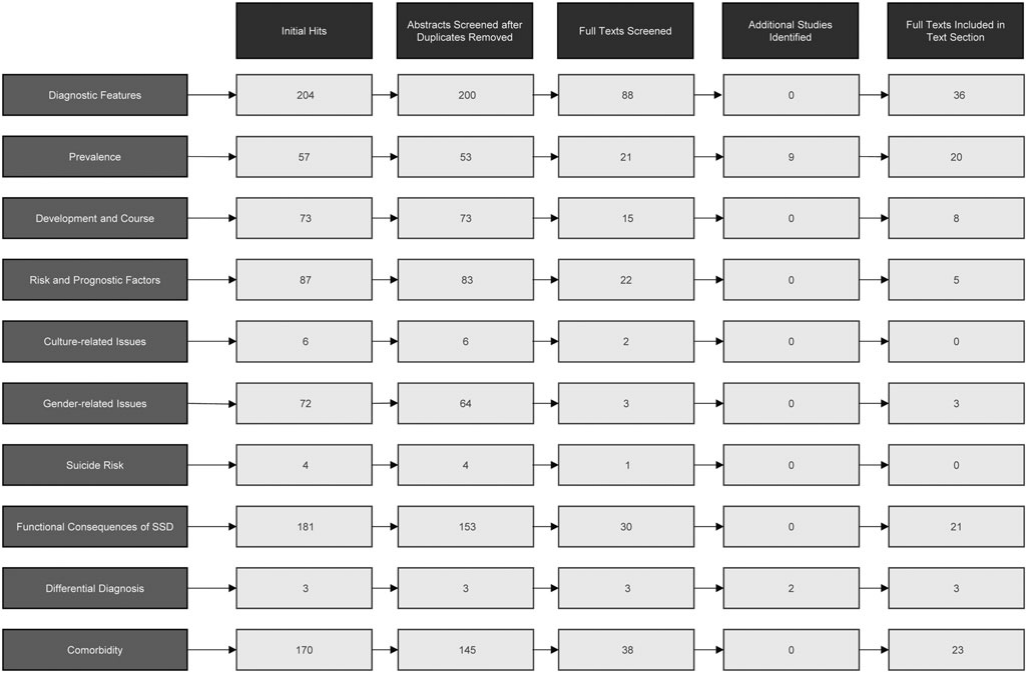
Study flow chart for scoping review. Displayed are the number of articles per DSM-5 text section. Some articles were identified for multiple text sections, thus the total number of articles included does not equal the column total (e.g. full texts included: column total, *n* = 119; total number of included articles, *n* = 59).

The literature search identified 882 articles. After duplicates were removed, 781 abstracts were screened for eligibility. After the screening of abstracts and including additional studies identified in the reference lists, full texts were screened in 250 studies. Several studies were identified for multiple DSM-5 text sections, and after full-text screening and excluding duplicate articles that were identified for multiple text sections eventually 59 articles were included into the analyses (see Fig. 1 for flow chart). Table 2 provides an overview of the included full texts with study design, sample size and operationalization of SSD criteria.

**Table 2.** Overview of included studies

Authors, year (Locations)	N	Population	Study design	Assessment of SSD	DSM-5 text section
Ahn et al., 2017 (Republic of Korea)	15/ 15/ 15	Female patients with SSD/Depression/ Healthy controls	Cross-sectional	Structured clinical interview diagnosed independently by two psychiatrists (SCID-5-CV)	Diagnostic Features
Axelsson et al., 2016 (Sweden)	52/ 52	Patients with health anxiety/ Healthy controls	Cross-sectional	Structured clinical interview (MINI, HPDI) with additional judgment by an assessor blind to previous diagnoses	Diagnostic Features, Differential Diagnosis
Bailer et al., 2016 (Germany)	200	Outpatients with SSD/ IAD/ Depression/ Healthy controls	Case–control	Self-report questionnaires (SHAI ⩾ 15, WI ⩾ 8) + Structured clinical interview based on DSM-5 research criteria	Diagnostic Features, Differential Diagnosis, Comorbidity
Bizzi et al., 2015 (Italy)	20/ 20	Inpatients, 8 to 15 years old with SSD and Disruptive Behavior Disorder	Cross-sectional	Clinical judgement based on DSM-5 SSD criteria	Diagnostic Features, Development and Course, Comorbidity
Bizzi et al., 2019 (Italy)	45/ 40/ 46	Inpatients, 8 to 15 years old, with SSD/ Disruptive Behavior Disorder/ Healthy controls	Cross-sectional	Clinical judgment after exclusion of organic origin in suspected SSD	Development and Course
Calabro et al., 2019 (Italy)	16	Female patients with Lipodystrophy	Cross-sectional	Psychiatric clinical assessment based on DSM-5 SSD criteria	Prevalence, Comorbidity
Cao et al., 2020 (China)	697	Patients from outpatient clinic	Cross-sectional	Semi structured clinical interview based on DSM-5 criteria (SCID-5-CV)	Diagnostic Features, Prevalence, Functional Consequences, Comorbidity, Gender-Related Diagnostic Issues
Carmassi et al., 2019 (Italy)	75	Patients in general practice	Cross-sectional	Semi structured clinical interview based on DSM-5 criteria (SCID-5-CV)	Functional Consequences
Claassen-van Dessel et al., 2016 (Netherlands)	325	Patients with medically unexplained symptoms	Cross-sectional	Self-report questionnaires (**A** PHQ-15 min. 2 items = bothered a lot, **B** WI ⩾ 6, **C** min. 1 symptom ⩾6 months)	Diagnostic Features, Prevalence, Functional Consequences, Comorbidity
Clarke et al., 2013 (USA, Canada)	46	Field trials	Cross-sectional	Structured clinical interview based on DSM-5 criteria (SCID-5-CV)	Diagnostic Features
Cozzi et al., 2017 (Italy)	306	Children 7–17 years who visited the pediatric emergency department with symptoms of predominant pain	Cross-sectional	Clinical judgment of medical records based on DSM-5 definition of SSD	Prevalence
Creed et al., 2012 (United Kingdom)	952/ 339/ 107	General population	Cross-sectional	Self-report questionnaires (SSI ⩾ 26)	Diagnostic Features, Prevalence
Dimsdale et al., 2013 (United States)	na	na	Review	na	Diagnostic Features, Prevalence
Eken et al., 2019 (Turkey)	19/ 21	Outpatients with SSD without history of neurological disorders or chronic general medical conditions/ Healthy controls	Cross-sectional	Clinical judgment oriented on DSM-5 SSD diagnostic criteria	Diagnostic Features
Fergus et al., 2019 (USA)	202	Primary care patients	Cross-sectional	Structured clinical interview (ADIS-5 SSD module), Self-report questionnaires (WI-6)	Prevalence, Differential Diagnosis, Comorbidity
Gan et al., 2016 (USA)	53/ 125	Patients with semantic dementia/ Alzheimers	Retrospective cohort	Clinical judgment oriented on DSM-5 SSD diagnostic criteria	Prevalence, Comorbidity
Gao et al., 2018 (Australia)	53	Adolescent patients 12 to 19 years with somatic disorders from Tertiary Children's Hospital	Retrospective cohort	Clinical judgment of medical recors oriented on DSM-5 SSD diagnostic criteria	Development and Course
Guidi et al., 2013 (Italy)	70	Patients with congestive heart failure	Cross-sectional	Ad hoc structured clinical interview based on DSM-5 SSD diagnostic criteria	Diagnostic Features, Prevalence, Functional Consequences, Comorbidity
Hatta et al., 2019 (Japan)	28/ 26	Adolescent inpatients 7 to 15 years with SSD/ Healthy controls	Case control	Clinical judgment oriented on DSM-5 SSD diagnostic criteria	Development and Course
Häuser et al., 2015 (Germany)	156	Outpatients with fibromyalgia where medical testing excluded somatic diseases fully explaining the symptoms	Cross-sectional	Self-report questionnaires (**A** PHQ-15 min. 1 item = bothered a lot, **B** WI ⩾ 6, **C** min. 1 symptom ⩾ 6 months)	Diagnostic Features, Prevalence, Functional Consequences, Comorbidity
Huang et al., 2016 (Taiwan)	471	Patients from psychiatric hospital	Cross-sectional	Structured clinical interview (diagnostic criteria DSM-5), Self-report questionnaires (**A** PHQ-15, **B** HAQ)	Diagnostic Features, Prevalence
Huang et al., 2017 (Taiwan)	168/ 106	Individuals with SSD/ Healthy controls	Cross-sectional	Clinical judgment oriented on DSM-5 SSD diagnostic criteria	Comorbidity
Huang and Liao, 2018 (Taiwan)	107	Psychiatric outpatients	Cross-sectional	Clinical judgment oriented on DSM-5 SSD diagnostic criteria	Diagnostic Features
Huang et al., 2019 (Taiwan)	53/ 52	Patients with SSD/ Healthy controls	Cross-sectional	Clinical judgment not further specified	Diagnostic Features, Comorbidity, Gender-related Diagnostic Issues
Hüsing et al., 2018 (Germany)	438	Patients from outpatient psychosomatic clinic	Cross-sectional	Structured clinical interview based on DSM-5 criteria (SCID-5-CV)	Diagnostic Features, Prevalence, Functional Consequences, Comorbidity
Inamura et al., 2015 (Japan)	40/ 21	Outpatients ⩾ 65 years with SSD without a physical disease capable of explaining somatic symptoms/ Healthy controls	Cross-sectional	Clinical judgment oriented on DSM-5 SSD diagnostic criteria	Development and Course
Kim et al., 2019 (Republic of Korea)	18/ 20	Patients with SSD/ Healthy controls	Cross-sectional	Structured clinical interview based on DSM-5 criteria (SCID-5)	Diagnostic Features
Klaus et al., 2015 (Germany)	321	General population	Prospective	Structured clinical interview for DSM-IV (SCID I) Self-report questionnaires (PHQ-15, B criteria items)	Diagnostic Features, Risk and Prognostic Factors, Functional Consequences
Klaus et al., 2017 (Germany)	28	Female patients with fibromyalgia	Ambulatory assessment	Self-report questionnaires (PHQ-15, 3 self-developed B criteria items 6 times daily for 14 days)	Diagnostic Features, Comorbidity
Kop et al., 2019 (Netherlands)	448	General population	Cross-sectional	Self-report questionnaires (SSD-12 ⩾15)	Functional Consequences, Comorbidity
Lee et al., 2015 (China)	3014	General population	Cross-sectional	Self-report questionnaires (PHQ-15 ⩾ 10, WI-5 ⩾ 4)	Functional Consequences
Lee et al., 2018 (South Korea)	23/ 20	Psychiatric outpatients with SSD/ Healthy controls	Cross-sectional	Clinical judgment oriented on DSM-5 SSD diagnostic criteria	Diagnostic Features
Lehmann et al., 2019 (Germany)	41	General practitioners	Focus group	na	Diagnostic Features
Li et al., 2016 (China)	11/ 12	Patients with SSD/ Healthy controls	Cross-sectional	Clinical judgment oriented on DSM-5 SSD diagnostic criteria	Diagnostic Features
Liao et al., 2019 (Taiwan)	107/ 100	Psychiatric outpatients with SSD/ Healthy controls	Cross-sectional	Clinical judgment oriented on DSM-5 SSD diagnostic criteria	Diagnostic Features, Functional Consequences, Comorbidity
Limburg et al., 2016 (Germany)	399	Outpatients with vertigo/ dizziness from a tertiary neurological care setting	Cross-sectional	Self-report questionnaires (**A** PHQ-15 min. 1 item = bothered a lot; **B** affective = WI ⩾ 6; **B** cognitive = CABAH subscales Autonomic Sensations ⩾ 5; Bodily Weakness ⩾ 8; **B** behavioral = SAIB; **C** min. 1 symptom ⩾ 6 months)	Diagnostic Features, Prevalence, Functional Consequences, Comorbidity
Limburg et al., 2017 (Germany)	239	Outpatients with vertigo/ dizziness from a tertiary neurological care setting	Prospective	Self-report questionnaires (**A** PHQ-15 min. 1 item = bothered a lot; **B** affective = WI ⩾ 6; **B** cognitive = CABAH subscales Autonomic Sensations ⩾ 5; Bodily Weakness ⩾ 8; **B** behavioral = SAIB; **C** min. 1 symptom ⩾ 6 months)	Diagnostic Features, Development and Course, Risk and Prognostic Factors, Comorbidity
Malas et al., 2018 (USA)	77	Outpatient pediatric primary care practitioners	Focus group	na	Diagnostic Features
Mander et al., 2017 (Germany)	84	Psychosomatic inpatiens	Prospective	Structured clinical interview for DSM-IV (SCID I)	Development and Course
Newby et al., 2017 (Australia)	118	Patients with SSD or IAD	Cross-sectional	Structured clinical interview (ADIS-5)	Diagnostic Features, Functional Consequences, Comorbidity
Orengul et al., 2020 (Turkey)	52/ 42	Children with psychogenic and functional breathing disorders/ Healthy controls	Cross-sectional	Semistructured diagnostic interview according to DSM-5 (Kiddie schedule)	Prevalence, Comorbidity
Regier et al., 2013 (USA, Canada)	not specified	Participants who registered with field trial center	Cross-sectional	Structured clinical interview based on DSM-5 criteria (SCID-5)	Diagnostic Features
Rief et al., 2011 (Germany)	154/ 167	General population with either high or low scores for somatic symptoms	Prospective	Self-report questionnaires (PHQ-15 ⩾ 5, WI-7), Structured clinical interview	Diagnostic Features, Prevalence, Functional Consequences
Schulte and Petermann, 2011 (Germany)	na	Children and adolescents	Review	na	Diagnostic Features
Schumacher et al., 2017 (Germany)	108/ 213	General population	Prospective	Self-report questionnaires (PHQ-15 ⩾ 5), Structured clinical interview oriented on DSM-5 SSD diagnostic criteria	Diagnostic Features, Risk and Prognostic Factors, Functional Consequences
Sirri and Fava, 2013 (Italy, USA)	na	na	Review	na	Diagnostic Features, Functional Consequences
Suzuki et al., 2017 (Japan)	214/ 104/ 197/ 742	Patients from Department of General Medicine with either probable or definite SSD/ matched or unmatched medical disease	Cross-sectional	Clinical judgment oriented on DSM-5 SSD diagnostic criteria	Prevalence
Tomenson et al., 2012 (United Kingdom)	609	Patients from general practices	Prospective (partially retrospective data)	Self-report questionnaires (SSI, WI)	Risk and Prognostic Factors
Toussaint et al., 2016 (Germany)	698	Psychosomatic outpatients	Cross-sectional	Self-report questionnaires (SSD-12)	Functional Consequences, Comorbidity
Toussaint et al., 2017 (Germany)	2362	General population	Cross-sectional	Self-report questionnaires (SSD-12)	Functional Consequences, Comorbidity
Toussaint et al., 2018 (Germany)	501	Primary care patients	Cross-sectional	Self-report questionnaires (SSD-12)	Comorbidity
Umemura et al., 2019 (Japan)	1202	Patients with orofacial pain who were reffered after organic dental/ oral disease was excluded	Cross-sectional	Structured clinical interview (diagnostic criteria DSM-5) Eher Clinical judgment oriented on DSM-5 SSD diagnostic criteria	Prevalence
van Eck van der Sluijs et al., 2017 (Netherlands)	187	Patients with SSD	Cross-sectional	Clinical judgment of patient files based on DSM-5 definition of SSD	Diagnostic Features, Comorbidity
van Geelen et al., 2015 (Sweden)	2476	General adolescent population	Cross-sectional	Self-report questionnaires (**A** PSP scale, **B** three items to assess psychological concerns)	Diagnostic Features, Prevalence, Development and Course, Gender-related Diagnostic Issues, Functional Consequences
Voigt et al., 2012 (Germany)	456	Psychosomatic inpatients	Prospective	Self-report questionnaires (**A** PHQ-15 min. 1 item = bothered a lot, **B** WI-14 ⩾ 6, SAIB, **C** min. 1 symptom ⩾ 6 months)	Diagnostic Features, Prevalence, Functional Consequences
Voigt et al., 2013 (Germany)	322	Psychosomatic inpatients	Prospective	Self-report questionnaires (**A** PHQ-15 min. 1 item = bothered a lot, **B** WI-14 ⩾ 6, SAIB, **C** min. 1 symptom ⩾ 6 months)	Diagnostic Features, Risk and Prognostic Factors, Functional Consequences
Walentynowicz et al., 2017 (Belgium)	30/ 24	Patients with SSD with medically unexplained dyspnea/ Healthy controls	Cross-sectional	Structured clinical interview (diagnostic criteria DSM-5) Eher: Structured clinical interview for DSM-IV (SCID I) + psychological SSD criteria based on DSM-5 definition of SSD	Diagnostic Features
Wollburg et al., 2013 (Germany)	230	Psychosomatic inpatients	Cross-sectional	Self-report questionnaires (**A** PHQ-15 min. 1 item = bothered a lot, **B** WI-14 ⩾ 6, SAIB, **C** min. 1 symptom ⩾ 6 months)	Diagnostic Features, Functional Consequences
Xiong et al., 2017 (China)	491	Patients from general hospital outpatient settings	Cross-sectional	Structured clinical interview (ICAB) Self-report questionnaires (**A** PHQ-15 min. 1 item = bothered a lot, **C** min. 1 symptom ⩾ 6 months)	Prevalence, Functional Consequences

*Note*. ADIS-5, Anxiety Disorders Interview for DSM-5; HAQ, Health Anxiety Questionnaire; HPDI, Health Preoccupation Diagnostic Interview; IAD, Illness Anxiety Disorder; ICAB, Interview about cognitive, affective, and behavioral features associated with somatic complaints; MINI, Mini-International Neuropsychiatric Interview 6; PHQ-15, Patient Health Questionnaire 15; PCS, Pain Catastrophizing Scale; PSP scale, Psychosomatic Problems Scale; SAIB, Scale for the Assessment of Illness Behavior; SCID, Structured Clinical Interview for Disorders according to DSM-5; SCL-90, Symptom Checklist; SSD, Somatic Symptom Disorder; SSD-12, Somatic Symptom Disorder B-criteria Scale; SHAI, Short Health Anxiety Inventory; SSI, Somatic Symptom Inventory; WI, Whitley Index;

### Diagnostic features

#### Reliability of the SSD criteria

In the DSM-5 field trials (Clarke et al., 2013), SSD showed good inter-rater reliability between clinicians of intra-class *κ* = 0.61 (Regier et al., 2013), which compared favorably with other psychiatric disorders (Dimsdale et al., 2013). One study indicated that SSD and IAD were more reliable diagnoses than the DSM-IV diagnosis of hypochondriasis (Newby, Hobbs, Mahoney, Wong, & Andrews, 2017). Another study indicated by an overall interrater agreement of Cohen's *κ* = 0.85 that clinicians could distinguish well between healthy controls and patients with SSD or IAD (Axelsson, Andersson, Ljotsson, Wallhed Finn, & Hedman, 2016). Agreement between raters was, however, much lower regarding severity, pain and persistence specifiers of SSD.

#### Validity of the SSD criteria

Studies in psychosomatic clinics showed a higher frequency for DSM-5 SSD compared to DSM-IV somatoform disorders (Hüsing, Löwe, & Toussaint, 2018; Voigt et al., 2012). However, mental impairment at discharge was greater for SSD compared to DSM-IV somatoform disorders (Voigt et al., 2012). A study in patients with medically unexplained symptoms also indicated more severe physical symptoms and impairment in patients with SSD compared to DSM-IV somatoform disorder (Claassen-van Dessel, van der Wouden, Dekker, & van der Horst, 2016). In contrast, in patients with dizziness and vertigo, lower impairment for patients with SSD compared to patients with DSM-IV somatoform disorders was observed (Limburg, Sattel, Radziej, & Lahmann, 2016). Rief and colleagues (Rief, Mewes, Martin, Glaesmer, & Braehler, 2011) concluded in their early evaluation of SSD that the SSD criteria themselves are not over-inclusive.

The majority of studies considered the inclusion of the B-criteria as a positive change in the diagnostic conception (Claassen-van Dessel et al., 2016; Klaus et al., 2015; Wollburg, Voigt, Braukhaus, Herzog, & Löwe, 2013). However, the choice of the three psychological B-criteria was criticized (Klaus et al., 2015), and the relevance of the clinical context and the interpretation of these criteria were highlighted for diagnosing SSD and its severity (Cao et al., 2020; Huang, Chen, Chang, & Liao, 2016). Two studies compared SSD and IAD, indicating higher health service use, more comorbid anxiety disorders (Bailer et al., 2016; Newby et al., 2017), more severe health anxiety, depression and somatic symptoms in individuals with SSD compared to individuals with IAD (Newby et al., 2017).

With regard to SSD in early life, SSD criteria were seen as helpful (van Geelen, Rydelius, & Hagquist, 2015) and more suitable for children and adolescents compared to prior diagnoses (Schulte & Petermann, 2011). Other authors suggested that an insecure and disorganized attachment style toward parents might be associated with adolescent SSD (Bizzi, Cavanna, Castellano, & Pace, 2015).

A general population study indicated that the total number of somatic symptoms in the general population was an independent predictor for health status (Creed et al., 2012). The authors concluded that these findings supported abandoning the diagnostic criterion that somatic complaints must be medically unexplained, as was required with somatoform disorders in DSM-IV and ICD-10. The predictive validity of SSD's diagnostic criteria was further demonstrated in psychosomatic inpatients (Voigt et al., 2013) and in patients with fibromyalgia (Klaus, Fischer, Doerr, Nater, & Mewes, 2017). Further, psychological distress was more strongly associated with patient complexity than the number of physical symptoms (van Eck van der Sluijs, de Vroege, van Manen, Rijnders, & van der Feltz-Cornelis, 2017). Another study indicated that patients with SSD showed a lower level of functioning and quality of life than healthy controls (Liao, Ma, Lin, & Huang, 2019). Comparing SSD to other diagnoses with regard to future healthcare utilization, SSD was found to be a valid, yet not a superior diagnosis (Schumacher, Rief, Klaus, Brähler, & Mewes, 2017). Note, however, that most of the studies mentioned had used proxy SSD criteria, otherwise specified “clinical judgment,” or previous diagnostic concepts for diagnosis. The diagnostic approaches used in each study are specified in Table 2.

#### Clinical utility of the new criteria

According to the DSM-5 field trials, SSD was among the most improved and useful criteria sets according to clinicians (Dimsdale et al., 2013; Regier et al., 2013). Other authors stressed the clinical utility of the new concept compared to DSM-IV (Voigt et al., 2012). Results from a qualitative study in general practitioners indicated that the advantages of SSD outweigh its disadvantages; especially the new psychological criteria and no longer making the diagnosis by exclusion of physical disease were regarded as improvements for clinical practice (Lehmann et al., 2019). In a study of pediatric primary care providers' experiences with patients with SSD, inexperience in applying the diagnostic criteria became apparent despite clinicians' postulated interest (Malas, Donohue, Cook, Leber, & Kullgren, 2018). In patients with fibromyalgia, clinical utility of SSD was judged to be limited (Häuser, Bialas, Welsch, & Wolfe, 2015). Two studies suggested using “diagnostic criteria for psychosomatic research” to improve DSM-5 diagnostic criteria (Huang & Liao, 2018; Sirri & Fava, 2013).

#### Associated features

Some potential additional features of SSD have been investigated, e.g., body scanning, illness denial, and self-concept of bodily weakness (Guidi, Rafanelli, Roncuzzi, Sirri, & Fava, 2013; Klaus et al., 2015; Wollburg et al., 2013). Other studies, all including small samples, suggested potential cerebral changes in patients with SSD, e,g. in the right temporal and the left inferior parietal gyri (Eken et al., 2019), frontostriatal circuit dysfunction (Ahn et al., 2017), changes in regional homogeneity values (Li et al., 2016), altered autonomic reactivity (Huang et al., 2019; Lee et al., 2018), altered pain processing (Kim, Hong, Min, & Han, 2019), and changes in autobiographical memories (Walentynowicz, Raes, Van Diest, & Van den Bergh, 2017). All of these studies were exploratory in nature.

### Prevalence

**Fig. 2.**
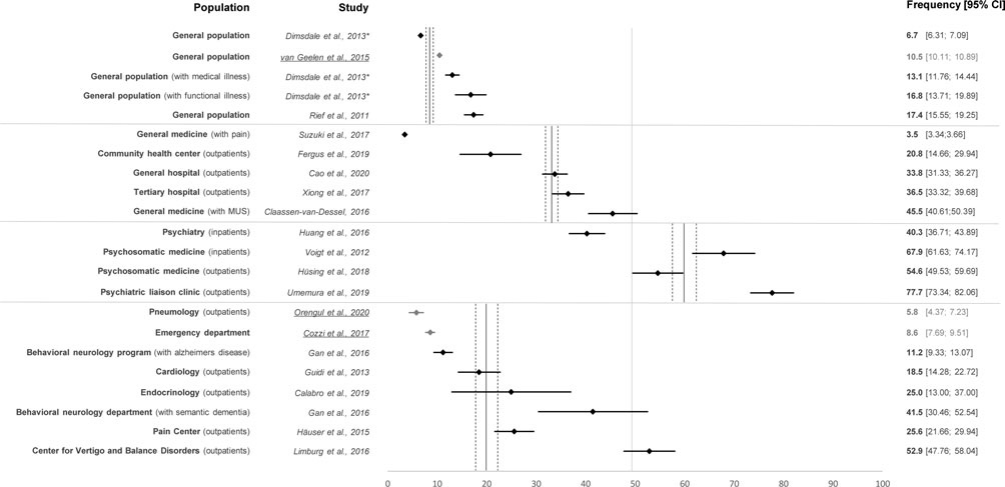
Forest plot of frequency estimates on somatic symptom disorder (with 95% CI). Of note, almost all frequencies estimates are based on proxy diagnoses of SSD, using self-report questionnaires or clinical judgement. The vertical lines indicate the mean values across several studies of a comparable setting (with 95% CI). Underlined references refer to studies in children and/or adolescents. *Data reported in this paper are based on Creed et al., 2012.

Only seven studies used semi-structured clinical interviews based on DSM-5 criteria to diagnose SSD (Calabro et al., 2019; Cao et al., 2020; Fergus, Kelley, & Griggs, 2019; Guidi et al., 2013; Huang et al., 2016; Hüsing et al., 2018; Umemura et al., 2019). Of these, Fergus et al., was conducted in primary care patients; the others were in specialized patient populations. All other studies used proxy-diagnosis operationalized by a combination of self-report questionnaires or by clinical determination of SSD. Prevalence studies in the general population using diagnostic criterion standard interviews are completely missing. Two studies reported data from randomly selected, adult, population-based samples using self-report questionnaires assessing previously considered SSD criteria (Creed et al., 2012; Dimsdale et al., 2013; Rief et al., 2011). SSD frequency rates in different medical and non-medical populations are summarized in Fig. 2. In general population studies, the frequency of proxy diagnosis for SSD varied between 6.7 and 17.4% [mean frequency 12.9% (95% confidence interval (CI) 12.5–13.3%)]. In studies conducted in non-specialized general medicine settings, frequency rates ranged from 3.5 (Suzuki, Ohira, Noda, & Ikusaka, 2017) to 45.5% [mean frequency 35% (95% CI 33.8–36.3%)] with the highest reported frequency rate in patients with medically unexplained symptoms. In diverse specialized care settings (e.g. pulmonology, cardiology, endocrinology, pain) including a pediatric emergency department (Cozzi et al., 2017), SSD frequency ranged between 5.8 and 52.9% [mean frequency 23.6% (95% CI 22.3–25%)]. The highest frequency of SSD was observed within mental health care settings specialized in SSD treatment with frequency rates ranging between 40.3 and 77.7% [mean frequency 60.1% (95% CI 57.8–62.4%)]. The current lack of studies examining the prevalence of SSD based on criterion-standard interviews is a major research gap, precluding reliable estimates of the prevalence of SSD.

### Development and course of SSD

#### Characteristics of adolescent SSD

A cross-sectional population-based study among adolescents (van Geelen et al., 2015) indicated that in those with serious psychological concerns regarding health and illness, reporting three or more persistent distressing somatic symptoms was significantly more common than reporting one or two. The most commonly reported somatic symptoms were unrefreshing sleep and headache, while the most commonly reported psychological symptoms were illness worries. Medical and psychiatric comorbidity was highest in the group reporting more than three somatic symptoms plus health/illness concerns.

A retrospective cohort study of adolescents admitted to a tertiary children's hospital with SSD or conversion disorder observed that 45% of the presenting symptoms were neurological and 39% involved pain (Gao, McSwiney, Court, Wiggins, & Sawyer, 2018). Two-thirds of adolescent SSD patients had at least one medical condition.

Bizzi and colleagues (Bizzi et al., 2015) compared inpatients with SSD *v.* disruptive behavior disorders and observed significant presence of insecure attachment in more than a half of the patients in both groups, but no significant differences between them in sociodemographic characteristics, attachment styles, or post-traumatic symptoms. The same group (Bizzi, Ensink, Borelli, Mora, & Cavanna, 2019) reported higher rates of insecure and disorganized attachment in school-aged children with SSD compared to healthy controls. Further, mentalization ability operationalized as reflective functioning was significantly lower in SSD children as compared to healthy controls. Another study (Hatta, Hosozawa, Tanaka, & Shimizu, 2019) observed no more traits of autism in adolescents with SSD than in healthy controls.

#### Characteristics of late-life SSD

A small cross-sectional study in late-life outpatients found that those with severe SSD had more cognitive impairment than those with milder SSD and healthy age-matched controls, but sampling bias prevents drawing reliable conclusions (Inamura et al., 2015).

#### Course of SSD

After inpatient treatment of adolescents with clinically diagnosed SSD, complete remission was observed in 49% (*n* = 18), response in 32% (*n* = 12), and no changes in 19% (*n* = 7) (Gao et al., 2018). Complete recovery after discharge was almost 20 times more likely in adolescents whose families fully accepted the SSD diagnosis compared to families with partial or no acceptance. Additionally, readmitted patients were eight times less likely to completely recover compared to first admission patients. In their 1-year prospective study of SSD in adult outpatients with vertigo and dizziness, a persistence rate of 82% and a remission rate of 18% was observed (Limburg et al., 2017). Finally, in a sample of SSD inpatients, ambivalent treatment motivation was related to more negative treatment outcomes (Mander et al., 2017).

### Risk and prognostic factors

#### Risk factors for SSD

A prospective study in patients with vertigo and dizziness (Limburg et al., 2017) found that those who developed SSD within the 1-year study period had higher baseline levels of health anxiety, were more catastrophizing, had a stronger self-concept of bodily weakness, showed more illness-related behaviors (e.g. taking medication), and had higher levels of depression and anxiety.

#### Prognostic factors for SSD remission

The same study showed that patients who recovered from SSD during the study period reported less catastrophizing at baseline compared with patients who did not recover (Limburg et al., 2017).

#### Prognostic factors for SSD associated outcomes

In a 4-year prospective general population study, SSD at baseline predicted the development of higher subjective impairment, health care utilization, and numbers of symptoms at 1-year and 4-year follow-ups (Schumacher et al., 2017). In a related prospective general population study, lower somatic symptom attribution and higher health anxiety were predictors of the number of medically unexplained symptoms after 4 years, while psychological variables did not predict impairment (Klaus et al., 2015). SSD at admission predicted poorer physical and mental functioning at 1-year follow-up in an inpatient sample with anxiety, depression, or somatoform disorders (Voigt et al., 2013). Additional predictors of limited physical functioning were a self-concept of bodily weakness, intolerance of bodily complaints, poor health habits, and somatic illness attributions. Finally, another prospective study indicated that the number of somatic symptoms and health anxiety were predictors of health care use 1 year later (Tomenson et al., 2012).

### Culture-related diagnostic issues

Although international studies of functional syndromes exist, no study could be identified that applied the DSM-5 criteria for SSD and examined them in a transcultural comparison.

### Gender-related diagnostic issues

A cross-sectional adolescent general population study reported that significantly more girls than boys reported problems regarding persistent distressing somatic symptoms (van Geelen et al., 2015). However, in a large study in general hospital outpatients (Cao et al., 2020) no gender differences in the prevalence of SSD were reported. An experimental study (Huang et al., 2019) tested whether heart rate variability differentiated healthy controls from patients with SSD. Compared to women without SSD, women with SSD showed a greater decrease in vagal activity when viewing stimuli related to somatic distress. This effect was not found in men.

### Suicide risk

No studies could be considered for review.

### Functional consequences

#### Functional impairment compared to healthy controls

Consistently across all six clinical adult studies, patients with SSD reported higher levels of impairment in terms of lower quality of life and functioning, and higher disability compared to individuals not meeting SSD criteria (Cao et al., 2020; Carmassi et al., 2019; Claassen-van Dessel et al., 2016; Guidi et al., 2013; Häuser et al., 2015; Liao et al., 2019). In addition, higher health care use and disability in individuals with SSD compared to those without were reported in two studies examining the same general population sample (Rief et al., 2011; Schumacher et al., 2017). One study examining adolescents showed higher levels of functional impairment in SSD compared to healthy controls and individuals with somatic symptoms without psychological features (van Geelen et al., 2015).

#### Comparisons with former diagnostic classifications

Six studies compared functional consequences in SSD to former or other proposed classifications for persistent somatic symptoms. Comparing SSD with DSM-IV or ICD-10 somatoform disorders, two studies with psychosomatic outpatient and inpatient samples reported lower mental quality of life in SSD (Hüsing et al., 2018; Voigt et al., 2012; 2013). These studies found similar (Voigt et al., 2012; 2013) or higher (Hüsing et al., 2018) physical quality of life in SSD. No differences were found in health care use. Higher disability levels and health care use were reported in SSD compared to IAD (Newby et al., 2017).

#### Relation with SSD severity

Six studies examined the relationship between SSD severity and functional consequences. Consistently, they reported increasing levels of impairment, health care use and decreasing quality of life with increasing number and severity of B-criteria across psychosomatic and other secondary care settings and the general population (Limburg et al., 2016; Toussaint et al., 2016; Toussaint, Löwe, Braehler, & Jordan, 2017; Wollburg et al., 2013; Xiong et al., 2017). In particular, the Somatic Symptom Disorder B-Criteria Scale (SSD-12), a self-report questionnaire assessing SSD B-criteria, has been shown to be a valid predictor of quality of life and health care use (Kop, Toussaint, Mols, & Löwe, 2019; Toussaint et al., 2016, 2017).

#### Health anxiety and somatic symptom burden as predictors

A large prospective general population study found health anxiety and somatic symptom burden to be independently related to functional impairment (Lee, Creed, Ma, & Leung, 2015). Klaus et al. found somatic symptoms to be more relevant in predicting impairment and health care use than psychological features (Klaus et al., 2015).

### Differential diagnosis

#### Illness Anxiety Disorder (IAD)

Given that IAD is diagnosed when there are no or only minimally distressing persistent somatic symptoms, by DSM-5 definition, a patient can either be diagnosed with SSD or IAD, but not both (American Psychiatric Association, 2013). Nevertheless, recent research reported comorbidity of 8% of IAD and SSD (Fergus et al., 2019) and that different raters sometimes disagree in their diagnostic classification of IAD and SSD (Axelsson et al., 2016). Other authors have questioned the utility of distinguishing them at all, as the diagnoses may have more in common than sets them apart, as reported in a case-control study that observed no significant differences between IAD and SSD regarding health anxiety, illness behavior, somatic symptom attributions, and physical concerns (Bailer et al., 2016).

#### Panic Disorder

Panic disorder is indicated as a differential diagnosis in DSM-5, yet raters in an interview study disagreed whether panic disorder should be an additional diagnosis or whether panic symptoms were part of SSD (Axelsson et al., 2016).

### Comorbidity

#### Comorbidity with mental disorders

**Fig. 3.**
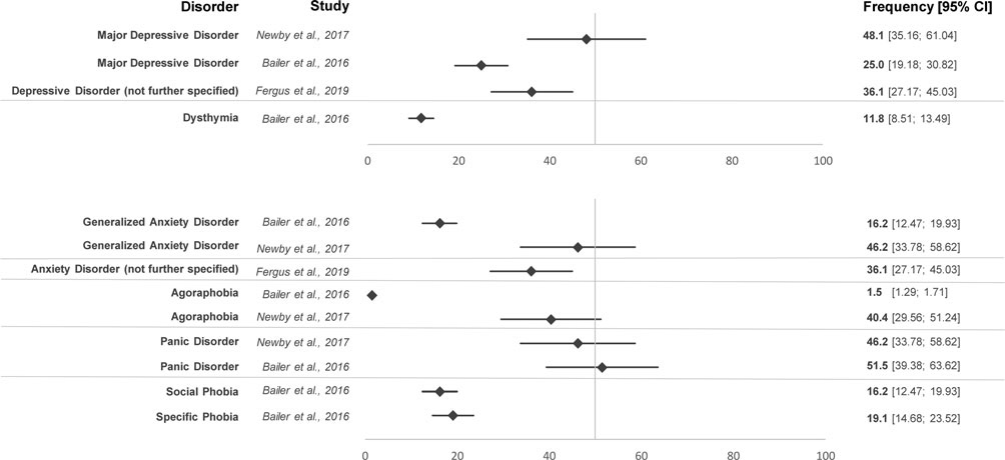
Forest plot of frequency rates of mental comorbidities in somatic symptom disorder (with 95% CI).

Most studies assessing other mental disorders or self-reported psychopathological symptoms in patients with SSD found high comorbidity rates with depression and anxiety whereas other psychiatric comorbidities were rarely assessed. The three studies shown in Fig. 3 investigated mental comorbidities in specific clinical outpatient samples with diagnostic interviews (Bailer et al., 2016; Fergus et al., 2019; Newby et al., 2017) (see Fig. 3).

In another general hospital outpatient sample, patients with SSD showed higher depression and anxiety levels than patients without SSD (Cao et al., 2020). Four studies observed higher self-reported depression and anxiety rates in patients with SSD compared to healthy controls (Bailer et al., 2016; Huang et al., 2017, 2019; Liao et al., 2019). Furthermore, three studies found significant associations between the SSD-12 score and depression and anxiety levels in the general population (Toussaint et al., 2017), in a primary care setting (Toussaint et al., 2018), and in a psychosomatic outpatient sample (Toussaint et al., 2016). Patients with SSD also showed higher depression (Claassen-van Dessel et al., 2016) and anxiety severity levels than patients with DSM-IV or ICD-10 somatoform disorders (Claassen-van Dessel et al., 2016; Hüsing et al., 2018).

SSD severity was not related to depression and anxiety severity in one study (Hüsing et al., 2018), while another (Claassen-van Dessel et al., 2016) found higher depression severity in moderate compared to mild SSD. Fergus and colleagues found that the severity of health anxiety was positively associated with the rates of medical and psychiatric comorbidity (Fergus et al., 2019). In another study, the complexity of SSD was associated with higher self-reported depression and anxiety (van Eck van der Sluijs et al., 2017). Comorbid depression was found to be associated with poorer overall functioning and quality of life (Liao et al., 2019). In a cross-sectional study of patients with vertigo and dizziness, the rate of psychiatric comorbidities was highest in SSD patients who fulfilled all three B-criteria (Limburg et al., 2016). Another study on this sample observed that comorbid depression and anxiety disorders were associated with the persistence of SSD (Limburg et al., 2017).

#### Comorbidity with physical conditions, including functional somatic syndromes

In studies examining different physical conditions, SSD criteria were met by 41.5% of patients with semantic dementia, 11.2% of patients with Alzheimer's disease (Gan, Lin, Samimi, & Mendez, 2016), 25% of female patients with non-HIV lipodystrophy (Calabro et al., 2019), and 18.5% of patients with congestive heart failure (Guidi et al., 2013). In individuals with fibromyalgia, 25.6% met SSD criteria (Häuser et al., 2015), and they had higher depression rates compared to fibromyalgia patients without SSD. In a smaller ambulatory study of fibromyalgia patients, 38.5% fulfilled the A- and B-criteria of SSD (Klaus et al., 2017). In a study assessing somatic conditions in patients with SSD, 28.8% had asthma, 23.1% had a circulatory condition, and 13.5% had gout, rheumatism or arthritis (Newby et al., 2017). In a population-based study, patients with different medical conditions scored higher on the SSD-12 compared to those free of these conditions, and the severity of the medical condition was associated with the SSD-12 score (Kop et al., 2019).

#### Comorbidity in children and adolescents

In a study in children and adolescents with SSD, no elevated levels of post-traumatic symptomatology were found (Bizzi et al., 2015). In a study including children with psychogenic and functional breathing disorders, 5.8% were diagnosed with persistent SSD (Orengul et al., 2020).

## Discussion

This scoping review summarizes the continuously growing scientific evidence regarding SSD after its introduction in DSM-5 in 2013 (American Psychiatric Association, 2013). Even though available research does not yet provide data on all DSM-5 text sections, it does allow a first assessment of the new diagnosis. Key research findings are discussed below and summarized together with the corresponding research gaps in Table 3.

**Table 3.** Key results of scoping review and resulting research gaps in the context of DSM-5 somatic symptom disorder

	Key results	Research gaps
Diagnostic features	Empirical evidence supports reliability, validity and clinical utility of the new SSD diagnostic criteria. The introduction of the psychological B-criteria was widely supported; however, further specification is called for. The same applies to the severity level and the use of the two specifiers (pain and persistence). Diagnostic coding of SSD and IAD as two separate diagnoses is not supported by the current evidence.	To further specify the existing B-criteria and to examine the inclusion of potential additional psychological criteria, To investigate overlap between affective, cognitive and behavioral facets of B-criteria, To evaluate the specifiers included in SSD diagnosis (predominant pain and/or a persistent course), To develop and evaluate diagnostic interviews to assess SSD, To develop diagnostic algorithms for the use of self-report questionnaires, To investigate the discriminant validity and clinical utility of the diagnostic distinction between SSD and IAD.
Prevalence	Only few studies investigated SSD frequency so far. Mean frequency of SSD was 12.9% (95% CI, 12.5 to 13.3) in the general population, 35% (95% CI, 33.8 to 36.3) in general medicine, and 23.6% (95% CI, 22.3 to 25.0) in specialized care. As these results are mainly based on self-report instruments or clinical assessments and partly investigate specific populations at high risk for SSD, it can be assumed that the actual SSD prevalence is significantly overestimated by these results.	To conduct prevalence studies in randomly selected, adult, population-based samples using criterion standard diagnostic interviews, To investigate SSD prevalence in different settings using criterion standard diagnostic interviews, stratified by severity level, age and gender.
Development and course	Empirical evidence indicates that adolescent SSD is characterized by multiple symptoms and illness worries. Overlap with medical conditions is high. Adolescent SSD remission rates after inpatient treatment appear promising, particularly if parents accept the diagnosis.	To investigate the natural course of SSD, To identify the age of onset for SSD, To identify risk factors for chronic courses of SSD, To identify mechanisms of symptom persistence in SSD, To investigate SSD specifics in children, adolescents and elderly.
Risk and prognostic factors	Psychological features of SSD, e.g., illness worries, catastrophizing, self-concept of bodily weakness, intolerance of bodily complaints, and somatic illness attributions were identified as risk factors for SSD development. SSD diagnosis itself was identified as a predictor of future functional impairment.	To cross-validate identified risk factors in independent studies, To identify additional risk factors for SSD development, remission and SSD related outcomes using prospective study designs, To promote research on prognostic factors for the course of SSD, To develop mechanism-based treatments based on identified risk factors and mechanisms.
Culture	Studies on cultural-related diagnostic issues in SSD are lacking so far.	To conduct comparative studies between different cultures, To examine the influence of acculturation on SSD diagnosis, To investigate culture-related diagnostic issues.
Gender	Studies on gender-related diagnostic issues in SSD are lacking so far.	To investigate gender-related factors in SSD, To examine gender differences relating to SSD diagnosis.
Suicide risk	Studies on suicide risk in SSD are lacking so far.	To investigate the prevalence of suicide and suicide risk in individuals with SSD.
Functional consequences	All included studies consistently show increased levels of functional impairment and disability and reduced quality of life in patients with SSD. Inconsistent results were reported with regard to health care use of patients with SSD. SSD severity and number of fulfilled of B-criteria was associated with increased functional impairment. Compared to former classifications, SSD patients reported lower mental health-related quality of life. Results regarding physical health-related quality of life are inconsistent.	To investigate the relative impact of somatic symptom burden versus psychological features on functional consequences, To investigate the influence of treatments on functional consequences in SSD.
Differential diagnosis	Illness anxiety disorder and panic disorder were discussed as a differential diagnosis to SSD, with the positions ranging on a spectrum between mutual exclusion and possible comorbidity.	• To compare predictive validity of the diagnoses in terms of treatment outcome and functional impairment.
Comorbidity	In adult SSD, evidence suggests high comorbidity rates with depressive disorders and anxiety disorders, while evidence for other mental disorders is scarce. SSD seems also to be associated with physical conditions and functional somatic disorders.	To disentangle associations between B-criteria and comorbidity with anxiety and depression, To improve understanding regarding SSD specifics in patients with comorbid physical diseases, To improve understanding of the relationship between SSD and other mental and physical conditions in children and adolescents.

### Reliability, validity, clinical utility, functional consequences

When a new diagnosis is introduced, it is expected to surpass its predecessor, especially in terms of reliability and validity. In the case of SSD, the available data demonstrate that it has better reliability and validity than its predecessor diagnoses. With regard to reliability, an acceptable to good interrater-reliability can be assumed for SSD, especially in comparison with other mental disorders (Dimsdale et al., 2013). The findings that patients with SSD display higher disability and lower health-related quality of life compared to the general population (Cao et al., 2020; Claassen-van Dessel et al., 2016; Huang et al., 2016) also support construct validity, as do the findings that with increasing severity of SSD, functional impairment also increases (Toussaint et al., 2016; Wollburg et al., 2013). Notably, the group of individuals identified by the new diagnosis of SSD is not identical to the group described by the previous diagnosis of somatization disorder. Regarding severity, it appears that SSD includes more severe cases in terms of mental quality of life, and perhaps somewhat milder cases in terms of physical quality of life in comparison to former classifications (Hüsing et al., 2018; Voigt et al., 2012). These results are not surprising considering that the new B-criteria emphasize psychological burden rather than somatic symptom count. Initial studies also indicate improved acceptance and clinical usefulness of SSD compared to its predecessor, which is most likely due to the added psychological criteria (B-criteria) and the removal of the need for medical diagnosis exclusion. However, the diagnostic distinction between SSD and IAD remains questionable: Based on present findings, IAD might be considered a milder form of SSD (Bailer et al., 2016).

### Individual diagnostic criteria

Our review yielded mixed results regarding the reliability of the B-criteria (Axelsson et al., 2016; Regier et al., 2013; Rief & Martin, 2014), and several alternative psychological features have been proposed (Martin & Rief, 2011). The empirically justified abolition of the medical inexplicability of the somatic symptoms was generally well-received by the clinical and scientific communities. Further, the diagnostic specifiers included in the diagnosis (predominant pain, persistence, and severity) were addressed in only a few studies (Axelsson et al., 2016; Katz, Rosenbloom, & Fashler, 2015; Rief & Martin, 2014). Study results on biomarkers of SSD are unlikely to be included in the diagnostic criteria in the near future due to their small sample sizes, exploratory nature, and rarely replicated results thus far. In summary, the greatest need for improvement of the SSD diagnostic criteria appears to be measurable and more precise diagnostic B-criteria. To date, it remains unclear how exactly the term “excessive” can be operationalized for symptom-related cognitions, anxiety, and behavior. A first attempt at operationalizing excessiveness can be found in a study (Toussaint, Hüsing, Kohlmann, Brähler, & Löwe, 2021), which describes that individuals with SSD spent an average of 4 h a day preoccupied with their somatic symptoms. Validated scales could also help operationalize cut-offs between normal and clinically abnormal range for the diagnostic B-criteria. Thus, a more precise definition of the content and cut-offs for the B-criteria definitely remains a task for the next edition of the DSM.

### Prevalence

Reliable data on the prevalence of SSD are still scarce. Only two studies that used proxy estimates of SSD, reported data from adult population-based surveys (Creed et al., 2012; Dimsdale et al., 2013; Rief et al., 2011) with frequency rates of 6.7 and 17.4% being higher than DSM-IV somatization disorder (~1–6%) (Creed & Barsky, 2004; Escobar, Burnam, Karno, Forsythe, & Golding, 1987), but lower than undifferentiated somatoform disorders (~20%) (Grabe et al., 2003). When making this comparison, it is important to keep in mind that the prevalence estimates for SSD are derived primarily from clinical assessments and self-report questionnaires, and not based on criterion standard diagnostic interviews. As self-report questionnaires and clinical assessment usually lead to an overestimation of the prevalence of mental disorders compared to diagnostic interviews, it can be assumed that the currently available data actually overestimate SSD prevalence. Since diagnoses in the available studies were based on self-report data, the mean 13% frequency of SSD in the general population indicates a considerable at-risk subgroup rather than a group with reliable diagnoses of SSD. All other studies included in this scoping review reported data from rather specific clinical settings, many of them limited by small samples sizes. In these studies, frequency rates covered a wide range from 3.5 to 77.7%. So far, none of the studies reported any prevalence data based on the SSD severity specifiers or age-adjusted and/or sex-adjusted prevalence estimates, or estimates stratified by age or gender. Studies in pediatric samples seem to find lower frequency rates. Our conclusions are tempered by the wide methodological heterogeneity of the included studies, i.e., different sample characteristics, sampling strategies, and varying diagnostic approaches. In conclusion, the concern that the new diagnosis of SSD might be overinclusive (Frances, 2013) cannot be completely dismissed, since the data on prevalence thus far are relatively unreliable. Accurate population-based estimates of SSD using criterion standard diagnostic interviews are needed in order to inform health care planning and resource allocation.

### Development, course, and risk factors

The evidence on SSD development, course and risk factors is unfortunately sparse and characterized by imprecise operationalization of SSD diagnosis often related to former diagnostic concepts. Whereas preliminary evidence on adolescent SSD suggests a frequent involvement of pain and illness worries in adolescent SSD along with promising remission rates in those who seek treatment (Gao et al., 2018; van Geelen et al., 2015), current evidence allows no conclusions on late-life SSD. In treated adult patients, remission rates appear considerably lower (Limburg et al., 2017) and influenced by interpersonal problems, somatic symptom severity and stress. Catastrophizing and the ideation of bodily weakness might be relevant aspects affecting SSD development and course (Limburg et al., 2017). However, to date, no study of SSD has yet investigated the age of onset, duration of untreated illness, natural course, risk factors for chronicity, or mechanisms of symptom persistence.

### Differential diagnosis and comorbidity

Differential diagnosis in SSD has rarely been investigated. Further research seems necessary to investigate how and if SSD and IAD differ from each other. Similar to DSM-IV somatization disorder (Kohlmann, Gierk, Hilbert, Brähler, & Löwe, 2016; Löwe et al., 2008), SSD frequently co-occurs with depressive and anxiety disorders. Reasons for the overlap of SSD, depressive disorders, and anxiety disorders may be partially overlapping diagnostic criteria, shared biological and psychological diathesis, the bidirectional risk for development of the other disorders, and a common basic construct (Löwe et al., 2008). Recent evidence also indicates that the SSD B-criteria are highly associated with depressive and anxiety symptoms (Hüsing et al., 2018; Kop et al., 2019; Toussaint et al., 2016, 2017). Thus, comorbidity rates of depression and anxiety may be higher in SSD compared to earlier diagnoses (Claassen-van Dessel et al., 2016; Hüsing et al., 2018). Moreover, the pattern of the B-criteria, the severity/complexity of SSD symptoms, and the number of fulfilled components (affective, cognitive, behavioral) seem to be associated with the frequency of comorbid mental disorders (Claassen-van Dessel et al., 2016; Fergus et al., 2019; Limburg et al., 2016; van Eck van der Sluijs et al., 2017). A variety of studies showed that among patients with various somatic diseases, roughly a quarter suffer from SSD. This suggests that it is important to consider the diagnosis of SSD in patients with somatic diseases in order to adequately treat them. Although no suitable reference could be identified, it should be mentioned that excessive somatic focus is a feature of both body dysmorphic disorder and SSD, but in the former, the patient is concerned with appearance, while in SSD the worry is about being ill.

### Further research gaps

Results of this scoping review indicate a large research gap regarding cultural- and gender-related aspects, as well as suicide risk in SSD (see also Table 3).

### Strengths and weaknesses

Results of the present scoping review must be interpreted in light of the following limitations. First, it is in the nature of a scoping review, that we could only report the results of studies that were found during our predefined literature search. Results may thus not completely reflect the state of knowledge on SSD; but rather the currently published state of knowledge. Second, the included studies were very heterogeneous in terms of study design, sample size, and operationalization of SSD diagnosis. In line with scoping review methods (Tricco et al., 2018), we did not conduct a formal quality assessment of all included research papers. Nevertheless, we ensured sufficient study quality by formulating strict requirements for study inclusion. Third, our search strategy was based on the predefined DSM-5 text sections. Other relevant aspects, e.g., the efficacy of treatments for SSD, were not considered and should be addressed in future reviews.

## Conclusion

Since the introduction of SSD in 2013, evidence has been accumulating that the new DSM-5 diagnosis appears to be reliable, valid and clinically useful. The introduction of positive psychological criteria and the elimination of the need to exclude medical explanations might have contributed to improved validity and acceptance. However, diagnostic changes in the ICD-11 (World Health Organization, 2021), other newly proposed classifications such as “functional somatic disorders” (Burton et al., 2020), and various other diagnostic conceptualizations for persistent somatic symptoms (Weigel et al., 2017) will require further scientific debate.

Thus far, it remains unclear how often the diagnoses of SSD or the respective ICD-11 diagnosis of BDD are actually used in different fields of medicine and countries (Kohlmann, Löwe, & Shedden-Mora, 2018). This is unfortunate because a missed diagnosis of SSD might prevent patients from receiving appropriate treatment. Valid self-report instruments for the diagnosis of SSD are available for the A-criterion, e.g., with the Somatic Symptom Severity Scale-8 (SSS-8) (Gierk et al., 2014) or the Patient Health Questionnaire-15 (PHQ-15) (Kroenke, Spitzer, Williams, & Löwe, 2010), and for the B-criteria, e.g., with the Somatic Symptom Disorder B-Criteria Scale (SSD-12) (Toussaint, Hüsing, Kohlmann, & Löwe, 2020). Beyond these instruments, the recommendations of the EURONET-SOMA group for assessing core outcome domains may help to improve the comparability of results from clinical trials in the future (Rief et al., 2017). In addition, research in recent years has led to a better understanding of the mechanisms underlying the perception and experience of persistent somatic symptoms (Henningsen et al., 2018). Continuing this research has the potential to lay the groundwork for the development of mechanism-based therapeutic approaches in the near future. We hope that the diagnosis of SSD will be appropriately used in patient care and intensively researched to increase the knowledge about SSD and fill the research gaps (Table 3).

## Supplementary material

For supplementary material accompanying this paper visit https://doi.org/10.1017/S0033291721004177. click here to view supplementary material
