# Supplementary material for: Somatic symptom disorder: a scoping review on the empirical evidence of a new diagnosis
Source: Psychol Med. 2021 Nov 15;52(4):632–48. doi: 10.1017/S0033291721004177 (PMC8961337; doi:10.1017/S0033291721004177)
Supplement: Supplementary file 1 [file S0033291721004177sup001.docx]

# Bernd Löwe et al.: “Somatic Symptom Disorder: A Scoping Review on the Empirical Evidence of a New Diagnosis”

**Literature search conducted in**

- **Pubmed** accessed via: https://www.ncbi.nlm.nih.gov/pubmed/
- **Psyndex:** accessed via https://www.sub.uni-hamburg.de/de/recherche/datenbank-informationssystem/detail/datenbank/86.html
- **Cochrane Library** accessed via https://www-1cochranelibrary-1com-10072e6q20ed1.emedien3.sub.uni-hamburg.de/advanced-search

**General search terms**

(somatic symptom disorder[Title/Abstract] OR somatic symptom disorders[Title/Abstract] OR somatic symptom distress[Title/Abstract]) AND ("2009/11/01"[PDat] : "2020/01/31"[PDat] AND (English[lang])

**Additional search terms** [Title/Abstract] **based on DSM-5 SSD sections**

***Diagnostic Features***

AND (diagnostic features [Title/Abstract] OR characteristics [Title/Abstract] OR diagnostic criteria [Title/Abstract] OR diagnosis, symptoms [Title/Abstract] OR clinical presentation [Title/Abstract])

***Prevalence***

AND (prevalence[Title/Abstract] OR frequency[Title/Abstract] OR incidence[Title/Abstract] OR epidemiology[Title/Abstract] OR epidemiological[Title/Abstract] OR occurence[Title/Abstract])

***Development and Course***

AND (age of onset [Title/Abstract] OR age at onset [Title/Abstract] OR course [Title/Abstract] OR development [Title/Abstract] OR childhood [Title/Abstract] OR children [Title/Abstract] OR adolescence [Title/Abstract] OR adolescents [Title/Abstract]) OR pathology [Title/Abstract] OR pathogenesis [Title/Abstract] OR aetiology [Title/Abstract] OR etiology [Title/Abstract])

***Risk and Prognostic Factors***

AND (risk factor*[Title/Abstract] OR predictor*[Title/Abstract] OR independent variable*[Title/Abstract] OR hazard*[Title/Abstract] OR threat*[Title/Abstract] OR exposition*[Title/Abstract] OR influence*[Title/Abstract] OR cause*[Title/Abstract] OR history*[Title/Abstract] OR prognosis*[Title/Abstract] OR prognostic factor*[Title/Abstract])

***Culture-Related Diagnostic Issues***

AND (culture [Title/Abstract] OR culture-bound syndrome [Title/Abstract] OR immigrants [Title/Abstract] OR Asian [Title/Abstract] OR Arabic [Title/Abstract] OR African [Title/Abstract] OR Australian [Title/Abstract] OR Pacific [Title/Abstract] OR Middle East [Title/Abstract] OR Latin America [Title/Abstract] OR South America [Title/Abstract] OR China [Title/Abstract] OR Russia [Title/Abstract] OR Western Europe [Title/Abstract] OR Eastern Europe [Title/Abstract])

***Gender-Related Diagnostic Issues***

AND (gender [Title/Abstract] OR sex [Title/Abstract] OR male [Title/Abstract] OR female [Title/Abstract] OR men [Title/Abstract] OR women [Title/Abstract])

***Suicide Risk***

AND (suicide[Title/Abstract] OR suicidal ideation[Title/Abstract] OR attempted suicide[Title/Abstract] OR suicidal[Title/Abstract])

***Functional Consequences of Somatic Symptom Disorder***

AND (consequence [Title/Abstract] OR disability [Title/Abstract] OR functioning [Title/Abstract] OR impairment [Title/Abstract] OR quality of life [Title/Abstract] OR health [Title/Abstract] OR morbidity[Title/Abstract] OR mortality[Title/Abstract])

***Differential Diagnosis***

AND (differential diagnosis [Title/Abstract] OR differentiation diagnostics [Title/Abstract] or differential diagnostics [Title/Abstract] or discriminatory diagnostics [Title/Abstract])

***Comorbidity***

AND (comorbidity [Title/Abstract] OR comorbid [Title/Abstract] OR multimorbidity [Title/Abstract] OR co-occuring [Title/Abstract] OR overlap [Title/Abstract] OR depression [Title/Abstract] OR anxiety [Title/Abstract] OR psychopathology [Title/Abstract])
